# Dr. James B. McCaw

**Published:** 1906-09

**Authors:** 


Dr. James B. McCaw, of Richmond, Va., died at his home in that city August 14, 1906, aged 84 years. He was a surgeon in the Confederate army and during the civil war had charge of Chimborazo Hospital, in Richmond, where 76,000 Confederate soldiers were treated. Dr. McCaw was the father of Surgeon Walter D. McCaw, United States Army, who was stationed at Buffalo for some years and who is now engaged in compiling the Index Catalogue of the library of the Surgeon General's office at Washington.

